# Properties and Environmental Impact of Cement Mortar Using Spodumene Mining Residue as Mineral Admixture

**DOI:** 10.3390/ma19040729

**Published:** 2026-02-13

**Authors:** Cheng Li, Xiaoying Li, Weiping Yan, Zhenhua Feng, Binbin Tang, Wei Zhang, Ping Jiang

**Affiliations:** 1 Sichuan Experimental Testing and Research Center of Natural Resources (Sichuan Nuclear Emergency Technical Support Center), Chengdu 610084, China; 2 Evaluation and Utilization of Strategic Rare Metals and Rare Earth Resource Key Laboratory of Sichuan Province, Chengdu 610081, China; 3 State Key Laboratory of Environment-Friendly Energy Materials, School of Materials and Chemistry, Southwest University of Science and Technology, Mianyang 621010, China; 4 Institute of Multipurpose Utilization of Mineral Resources, Chinese Academy of Geological Sciences, Chengdu 610041, China; 5 Technology Innovation Center for Comprehensive Utilization of Strategic Mineral Resources, Ministry of Natural Resources, Chengdu 610041, China

**Keywords:** spodumene mining residue, mineral admixture, cement mortar, environmental impact, interaction mechanism

## Abstract

The rapid development of the lithium battery industry resulted in a large accumulation of spodumene mining residue (SMR). This paper explored the feasibility of using SMR as mineral admixtures in cement mortar. The properties of cement mortar, including flexural strength, compressive strength, fluidity, hydration characteristics, and durability, were studied. The interaction mechanism between SMR and cement mortar had been explored using the Dinger–Funk model, isothermal calorimetry, X-Ray Diffraction (XRD), fourier Transform Infrared Spectroscopy (FTIR), and thermogravimetry (TG) methods. Additionally, the environmental impact of cement mortar was quantitatively evaluated by the life cycle assessment method. The results showed that, while the dosage of SMR was no more than 20 wt.% replaced cement, the flexural strength, compressive strength, and anti-carbonation and sulfate corrosion resistance properties of S2 and S3 cement mortar were similar to that of the blank group. After curing for 28 d, the compressive strength of S1, S2, and S3 were 44.2 MPa, 43.15 MPa, and 40.32 MPa, respectively. SMR powder could improve the workability and reduce the cumulative hydration heat of cement mortar, which confirmed its application potential in large-volume concrete projects. The appropriate content of SMR incorporation into cement mortar could improve the structure and properties of cement-based materials through particle filling, the induced nucleation effect, and the pozzolanic effect. In addition, the utilization of SMR reduced the environmental emissions and resource consumption of cement-based materials. Using 1 m^3^ cement mortar as an example, for every 10 wt.% increase in SMR powder replacing cement, the energy consumption, the emissions of CO_2_, CO, C_x_H_y_, NO_x_, SO_2_, dust, and resource consumption of cement mortar were decreased by approximately 342 MJ, 40 kg, 8.1 g, 5.55 g, 88.3 g, 5.24 g, 1.80 kg, and 74.3 kg, respectively. The research findings of this paper are expected to promote the resource utilization of SMR and reduce the carbon emissions of the building materials industry.

## 1. Introduction

In China, the development of lithium batteries and the new electric vehicle industry have significantly driven the demand for lithium-containing raw materials including lithium carbonate and lithium hydroxide [1,2]. Data from the Ministry of Natural Resources of the People’s Republic of China shows that lithium resources in China comprise lithium mines in salt lakes, spodumene, and lepidolite ores [3]. In 2025, a world-class spodumene-type lithium mineralization belt measuring 2800 km in length was discovered in the Sichuan region of China. About 56% of lithium resources in China are located in the Sichuan province, and the of the majority of these resources are spodumene. During the mining process of spodumene, a large amount of mining waste is often generated for the overburden layer in open-pit mining, the surrounding rock during underground mining, and associated useless rock. Although the amount of spodumene mining residue (SMR) varies depending on the mining area, in general, for every one ton of spodumene olivine mined, more than two tons of waste will be produced. In 2024, spodumene production in China was estimated to be approximately 9 million tons, which means that mining waste exceeded 18 million tons. This extensive mining activity has led to the accumulation of mining residues and posed threats such as land resource depletion and airborne dust that jeopardizes the health of nearby residents [4]. Consequently, resource utilization of SMR is a problem that must be settled urgently. Indeed, Professor Raymond Pierrehumbert (University of Oxford) stated unequivocally: “With regard to the climate crisis, yes, it’s time to panic” [5]. Facing climate change, China has proposed “carbon peaking and carbon neutrality goals”. The “China Construction Industry Carbon Peak and Carbon Neutrality Research Report (2024)” showed that the total carbon emissions from construction and construction activities in China amount to 5.13 billion tons of CO_2_, accounting for 48.3% of the country’s energy-related carbon emissions. Among this, the production and transportation stages of building materials account for 42.89%, and the operation stage of buildings accounts for 55.66%. As environmental issues become increasingly serious, there is a high demand for green development in the construction materials industry that has a significant impact on the environment. The partial or complete replacement of cement by solid waste in the preparation of cement-based materials is regarded as a typical approach to the greening of building materials. It is of great significance to confirm the application potential of SMR in cement-based materials.

At present, SMR has not been widely utilized in building materials. It is important to distinguish between SMR and lithium slag. Lithium slag is the byproduct of producing lithium carbonate through the sulfuric acid roasting of spodumene ore, but SMR is the byproduct of spodumene mining [6]. Lithium slag had a relatively high utilization rate for its high pozzolanic activity used in cement-based materials. However, spodumene is often associated with quartz, mica, pyroxene, or feldspar [7], resulting in a high content of silicon (usually exceeding 65 wt.%, measured by SiO_2_), which caused the relatively low activity and utilization of SMR. Currently, SMR is mainly used for lithium resource extraction [8], backfilling mined-out areas [9], and as the raw materials for ceramics [10]. The application of SMR in cement-based materials has been less studied. The silicon–aluminum–calcium component content of SMR is relatively high, which may possess the potential to be used in cement-based materials [11]. However, ensuring the performance of products while maximizing the utilization rate of SMR is the key issue that needs to be solved. Therefore, it is highly necessary to conduct basic research on the preparation, properties, and mechanism of action of cement-based materials containing SMR.

In this research, the mechanical properties, long-term durability, and environmental impact of cement mortar containing SMR powder are studied. The interaction mechanism between SMR and cement paste was investigated. The research results of this paper will provide a theoretical basis for the resource utilization of spodumene mining residue in the construction materials field. It is also expected to provide a reference for carbon emission reduction in the building materials industry.

## 2. Materials and Methods

### 2.1. Materials

**Figure 1:**
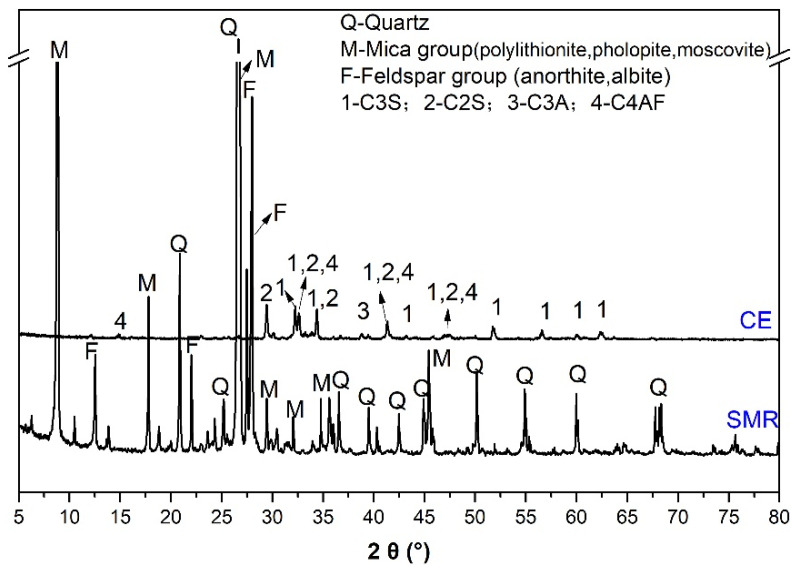
Mineral composition of raw materials [11].

**Figure 2:**
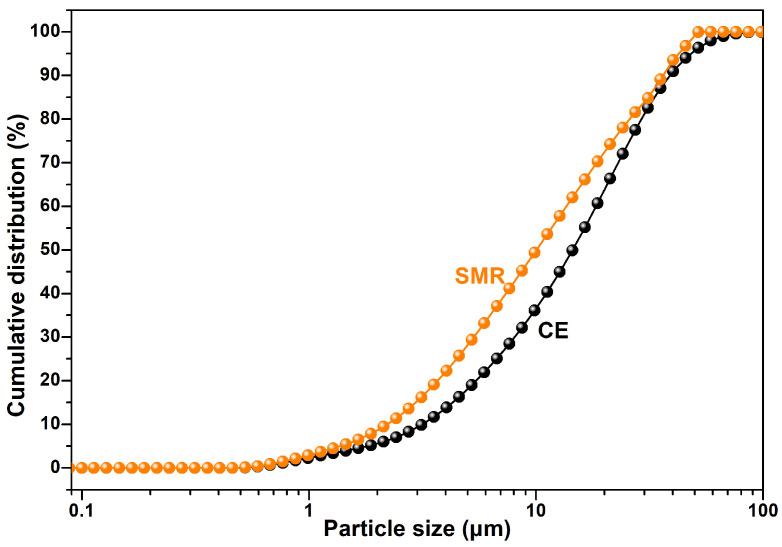
Cumulative distribution of cement and SMR powder.

**Figure 3:**
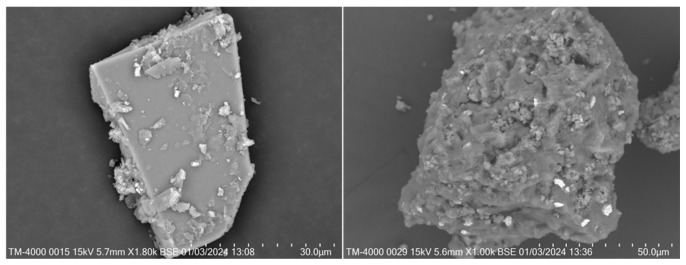
The particle morphology of SMR powder after grinding for 60 min.

In this research, cement (CE, P.O42.5, Lafarge cement Co., Ltd., Chengdu, China), spodumene mining residue (SMR, block, Garze Tibetan Autonomous Prefecture, China), and quartz sand (S, SiO_2_ ≥ 98%, China ISO standard sand, Xiamen ISO standard sand CO., LTD., Xiamen, China) were used as raw materials. The chemical composition and mineral composition of main raw materials are shown in Table 1 and Figure 1. From Figure 1, the mineral composition of SMR consisted of the mica group, feldspar group, and quartz, while that of cement included C_3_S, C_2_S, C_3_A, and C_4_AF. Before using SMR powder as a mineral admixture, the SMR block was ground in an M500 ball mill (M500, Jianyan Huazhao Instrument and Equipment Co., Ltd., Beijing, China) for 60 min with 0.05 wt.% triethanolamine (Purity > 99.0%, Lujia chemical Co., Ltd., Chengdu, China) as a grinding aid. The cumulative residue on a sieve of standard sand was tested by the sieving method and the results are listed in Table 2. The particle distribution of cement and SMR powders was tested by Master 3000 (Master 3000, Jianyan Huazhao Instrument and Equipment Co., Ltd., Beijing, China), and the results are shown in Figure 2. The particle size of cement is mainly distributed between 1 μm and 80 μm, while the particle size of SMR is mainly between 1 μm and 60 μm. The SMR particles were finer than the cement particles. The special surface area of CE and SMR were 392 m^2^/kg and 422 m^2^/kg, respectively, which were tested according to the Chinese standard GB/T 8074-2008 [12]. Figure 3 shows the particle morphology of SMR powder that was ground for 60 min; the particle shapes were irregular and the roundness was poor. According to the Chinese standard GB/T 1596-2017 [13], the strength activity index of SMR was tested, and the result was 71.5%, which indicated the low reactivity of SMR in cement-based materials.

The radionuclide of SMR was evaluated according to the Chinese standard GB 6566-2010 [14]. The results showed that the internal exposure index I_Ra_ was 0.3 and the external exposure index I_r_ was 0.5, which was within the range required by the standard. That is, SMR could be used as the main building material.

### 2.2. Mixing Proportion and Preparation

Table 3 lists the mixing proportion and curing condition of cement mortar containing SMR as a mineral admixture. The preparation, curing, and property testing of the cement mortar were conducted according to the Chinese standard GB/T 17671-2021 [15].

### 2.3. Methods

During the experimental process, all the mixed proportions of cementitious materials and cement mortar were based on Table 3. The water requirement of standard consistency and setting time of cementitious materials were tested according to the Chinese standard GB/T1346-2024 [16]. According to the Chinese standard GB/T 17671-2021 [14], cement mortar cube specimens (40 mm × 40 mm × 160 mm) were prepared, and the compressive strength and flexural strength were tested after standard curing of specimens for 3 d, 7 d, and 28 d. The flexural strength was tested under the loading rate of 50 ± 10 N/s [14]. The compressive strength was tested the under the loading rate of 2400 ± 200 N/s [14]. Prior to sample molding, the fluidity of the cement mortar was tested according to the Chinese standard GB/T2419-2005 [17]. After standard curing for 28 d and then drying for 48 h at 60 °C, the carbonation resistance property of the cement mortar was tested according to the Chinese standard GB/T 42277-2022 [18]. During the test conditions, carbon dioxide concentration, humidity, and temperature were set at 20%, 70%, and 20 °C, respectively. After standard curing for 28 d and then drying for 48 h at 80 °C, the resistance property of sulfate was evaluated according to the Chinese standard GB/T 50082-2024 [19], and the test cycle was set to 50 times.

Isothermal calorimetry (TAM AIRO8, TA Instrument Company, New Castle, DE, USA) was used to investigate the hydration heat characteristics of cementitious materials with or without SMR. We weighed 6 g of solid materials, including cement and SMR powder, according to Table 3. Then we poured 3 g of water into a test bottle, mixed manually for 1 min, and then tested at 20 °C for 7 days.

The simulation pore solution method was used to analyze the pozzolanic effect of SMR in cement mortar. Simulation pore solution was prepared using 0.001 mol/L Ca(OH)_2_ (Purity > 98.0%, Lujia chemical co.,Ltd, Chengdu, China) and 0.6 mol/L NaOH (Purity > 99.7%, Lujia chemical co.,Ltd, Chengdu, China). The pH of the simulation pore solution was controlled to approximately 13.6. SMR was soaked in the simulation pore solution at 20 °C to study the interaction between SMR particles and cement. After soaking for 48 h, a seemingly white flocculent product generated and attached to the surface of SMR particles. The white flocculent product was conducted using centrifugation, cleaned by self-made ultrapure water, and dried through a vacuum drying oven (DZF-6034, Yiheng Scientific Instruments Co., Ltd., Shanghai, China). Then XRD, FTIR, and the TG method was used to analyze the composition and microstructure of the white flocculent product.

The chemical composition of materials was tested through X-ray fluorescence analysis (XRF, Axios, PANalyti-cal B.V., Almelo, The Netherlands) with the maximum power, element range, and concentration range of 2.4 kW, fluorine to uranium, and 0.01% to 100%, respectively. The mineral composition of raw materials and samples were tested by X-ray diffraction analysis (XRD, DMAX1400, Rigaku Corporation, Tokyo, Japan) with the scanning range, scanning speed, and step size of 3–80° (2 theta), 8°/min, and 0.02°, respectively. Based on crystallography databases and inorganic crystal structure databases, XRD patterns were analyzed. Thermogravimetric analysis (TG) was used to test the weight loss of samples during the heating process. We weighed (12 ± 3 mg) samples, placed them in a corundum crucible, put them in the analyzer (STA449F5, Netzsch Instrument Manufacturing Co., Ltd., Germany), and then tested them from 30 °C to 1000 °C with a 20 °C/min heating rate under a nitrogen atmosphere. Fourier transform infrared (FTIR) spectroscopy analysis was used to test the chemical groups or chemical bonds of samples. We weighed approximately 0.5 mg of white flocculent product with 1 mg of KBr, grinding until there were no visible particles. Then we molded the samples by compression and placed them into the analyzer (Spectrum One, PerkinElmer Instruments Co., Ltd., America). The testing range of this infrared testing instrument is from 400 cm^−1^ to 4000 cm^−1^, and the highest resolution is 0.5 cm^−1^.

## 3. Results and Discussion

### 3.1. The Properties of Cement Mortar Using SMR as Mineral Admixture

#### 3.1.1. The Physical Properties of Cement Mortar with SMR Powder as Mineral Admixture

**Figure 4:**
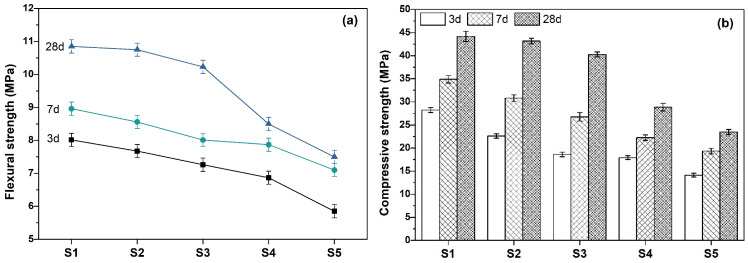
Mechanical properties of cement mortar with or without SMR as mineral admixture (**a**) flexural strength (**b**) compressive strength.

Figure 4a,b shows the flexural and compressive strengths of cement mortar, respectively. S1 was the blank group without SMR. With the curing age increased, the mechanical properties of cement mortar were enhanced, which was attributed to the increase in hydration products and the increasingly compacted microstructure of cement mortar. With the increase in SMR content, the mechanical properties of cement mortar were reduced due to the low activity of SMR. However, the mechanical properties of S2 and S3 after curing for 28 d were close to that of S1. In other words, when the content of SMR was less than or equal to 20 wt%, the long-term strength of S2 and S3 cement mortar were relatively excellent.

**Figure 5:**
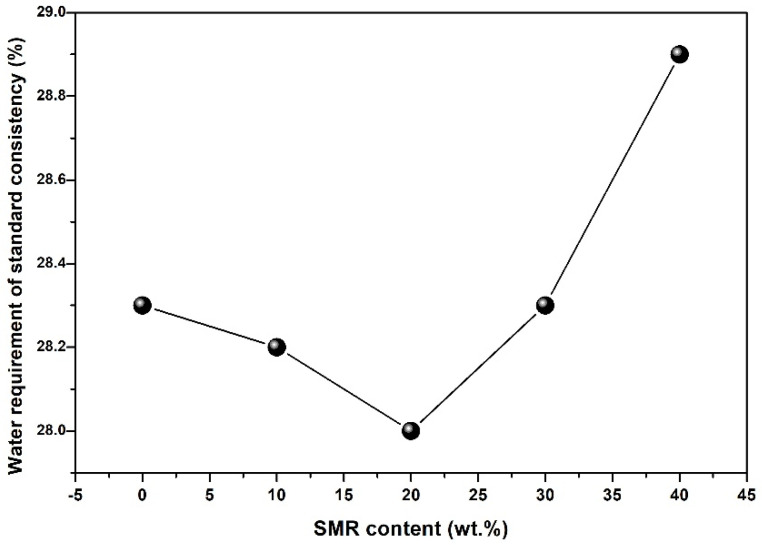
The water requirement of standard consistency of cementitious materials with SMR.

Figure 5 shows the water requirement of standard consistency of the binder with SMR powder partly replacing cement. With the content of SMR increased from 0 wt.% to 20 wt.%, the water requirement of standard consistency of the binder decreased. While the replacement content of SMR was 20 wt.%, the minimum water requirement of standard consistency was 28%. The result of mechanical properties of cement mortar with SMR showed that the SMR had poor pozzolanic activity. Meanwhile, the filling effect, grading adjustment function, and crystal nucleus induction effects of SMR were still capable of functioning effectively in cement-based materials. The finer SMR particles could fill the gaps between cement particles, thereby increasing the free water content in paste and enhancing the fluidity of the paste. Therefore, when the content of SMR increased from 0% to 20%, the water requirement of standard consistency of the binder decreased. However, due to the larger specific surface area of SMR particles, as the content of SMR further increased, the water consumption of the binder increased.

**Figure 6:**
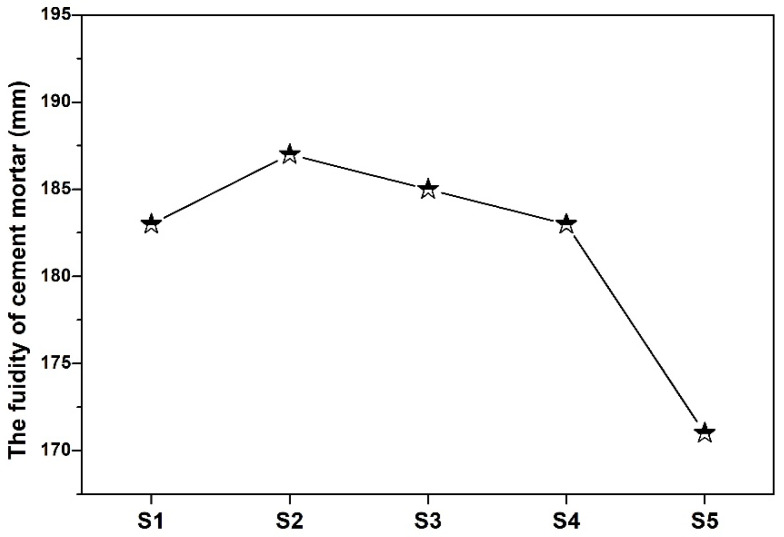
The fluidity of cement mortar with or without SMR.

Figure 6 shows the fluidity of cement mortar with or without SMR. Among all the cement mortars, S2 had the highest fluidity. Moreover, the fluidity of the mortars in both S2 and S3 was slightly higher than that of S1. When the content of SMR powder was lower than wt.%, more free water could be released into the paste due to better filling of the powder system, resulting in improved overall fluidity of cement mortar. However, while the SMR content was further increased, the water requirement of the paste increased due to the increase in fine powder; then, the fluidity of the cement mortar gradually decreased under the same water addition. The water requirement of standard consistency of cementitious materials confirmed the viewpoint.

#### 3.1.2. Hydration Characteristic of Cementitious Materials with SMR as Mineral Admixture

**Figure 7:**
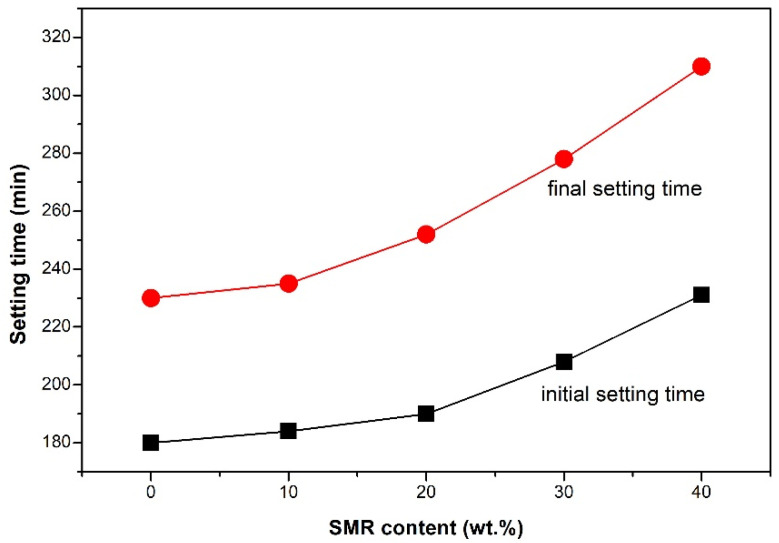
The setting time of cementitious materials with SMR powder.

**Figure 8:**
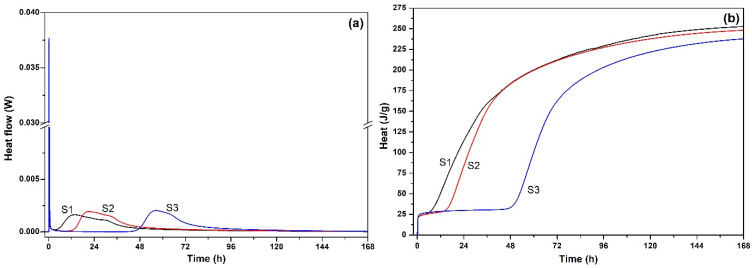
The heat and heat flow of cementitious materials with SMR as admixture. (**a**) heat flow of cementitious materials (**b**) cumulative heat release of cementitious materials.

In this research, the setting time and hydration heat release characteristics of cementitious materials with or without SMR were studied, and the results are shown in Figure 7 and Figure 8.

In Figure 7, under the same water addition, and with the increase of SMR powder, the initial setting time and final setting time of cementitious materials were prolonged. The longer setting time of the mortar with SMR powder also resulted in its lower early strength that was previously discussed. However, it was also confirmed that SMR was expected to reduce the heat release during the early hydration of cement-based materials, which was beneficial to the pouring of large-volume concrete to a certain extent. To verify our assumption, the early hydration heat release characteristics of the cement paste with SMR were further tested.

Figure 8a,b shows the heat flow and cumulative heat release of cement in 7 d, respectively. In Figure 8a, SMR powder did not change the hydration stage of the cement paste. The hydration process of all cement paste went through four stages: the hydration induction period, the hydration acceleration period, the hydration stabilization period, and the hydration deceleration period. However, with the content of SMR increased, the heat flow peaks of cement pastes were delayed, and the cumulative heat release of cement paste was reduced (in Figure 8b). Compared with the pure cement paste, the 7 d cumulative heat release of the cement paste with 20 wt.% SMR was reduced to about 223 J/g, which basically conformed to the heat release of medium-heat Portland cement.

#### 3.1.3. The Anti-Carbonation and Sulfate Corrosion Resistance Properties of Cement Mortar

**Figure 9:**
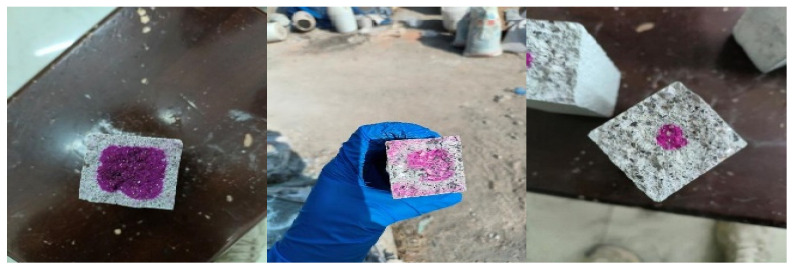
Test process of carbonation depth.

**Figure 10:**
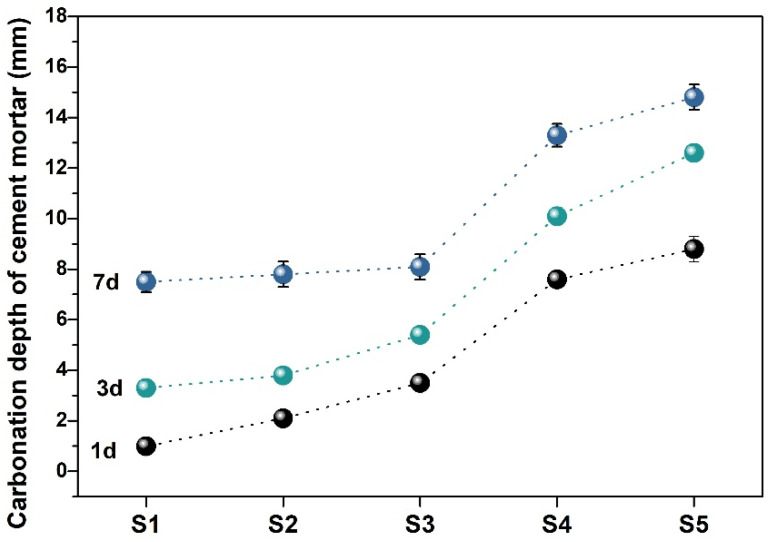
Carbonation depth of the cement mortar containing SMR.

Figure 9 and Figure 10 show the test process and result of carbonation depth of the cement mortar with SMR mineral admixture. S1 exhibited the strongest anti-carbonation performance and the slowest carbonation rate at the same age. The higher the content of SMR mineral admixture, the deeper the carbonation depth of the cement mortar. However, while the content of SMR was less or equal to 20 wt.%, the carbonation depth of the cement mortar samples was similar to that of S1.

**Figure 11:**
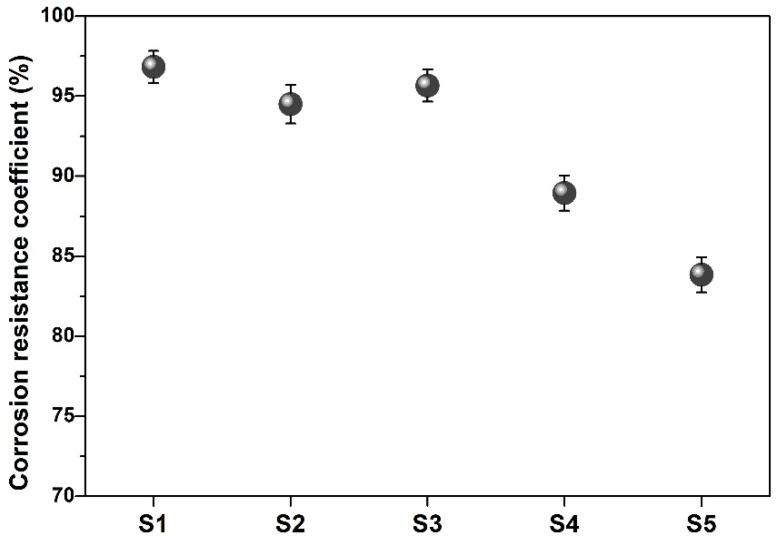
Corrosion resistance coefficient against sulfate of cement mortar.

Figure 11 shows the corrosion resistance coefficient of the cement mortar with or without SMR after 50 cycles of sulphate corrosion. While the content of SMR powder was less than 20 wt.%, the corrosion resistance coefficient of S1, S2, and S3 cement mortars was more than 93%, and there was no obvious damage on the surface of these samples, which were considered to have excellent resistance to sulfate corrosion. However, with the further increase of SMR powder, the crack of samples became more serious together, with fractures and surface peeling occurring.

Based on the results of both carbonation resistance and sulfate corrosion resistance, it was not hard to conclude that the addition of a small amount of SMR powder could densify the cement mortar structure and prevent the invasion of harmful ions. Nevertheless, the interaction mechanism between SMR powder and the cement paste remained elucidated.

### 3.2. Interaction Mechanism of SMR Mineral Admixture in Cement Mortar

Mineral admixtures often improved the microstructure and properties of cement-based materials through the filling effect [20], the induced nucleation effect [21], and the pozzolanic effect [22]. In this research, the interaction mechanism of SMR mineral admixture in cement mortar was studied and is discussed below.

#### 3.2.1. The Filling Effect of SMR Powder in Cement Mortar

For continuous particle size systems, the mathematical models of the most compact packing include Fuller’s compact packing theory and Andreasen’s classical continuous packing theory. Dinger and Funk modified the Andreasen equation by introducing a limited small particle size into the powder, resulting in the Dinger–Funk equation, as follows.

The model of Andreasen,

(1) $${U(D}_{P})=100{\left(\frac{D_{p}}{D_{max}}\right)}^{n}$$

The model of Dinger-Funk [23],

(2) $${U(D}_{P})=100\frac{D_{p}^{n}-D_{min}^{n}}{D_{mas}^{n}-D_{min}^{n}}$$

where ${U(D}_{P})$ is the cumulative percentage of particles with a diameter of D_p_ that pass through the sieve, %; D_p_ is the particle size of powder, μm; D_max_ is the diameter of the largest particle, μm; D_min_ is the diameter of the smallest particle, μm; and n is the distribution index.

The value of n was related to the proportion of fine particles in the particle system, and when n was within the range of 0.25 to 0.30, the optimal packing density could be achieved. We set n at 0.25, for the powders were all fine particles in this research. The particle size distribution determined by the Dinger–Funk mathematical model (DF model curve) should be the most excellent packing curve of particle groups. That is, the closer the particle size distribution of the particle group was to the DF curve, the denser the packing of that particle group would be.

**Figure 12:**
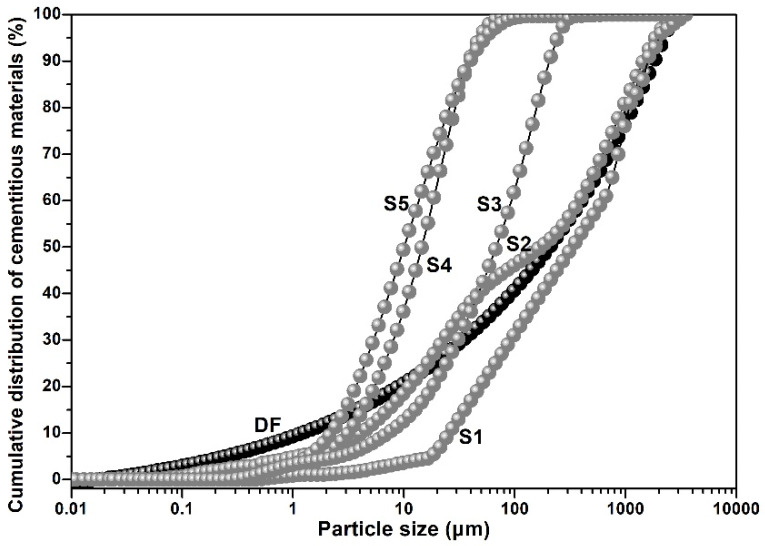
The cumulative distribution of cementitious materials particle groups.

Figure 12 shows the cumulative distribution of the cementitious materials particle groups of S1, S2, S3, S4, and S5. It was not hard to see that the cumulative distribution of cementitious materials of the S2 group was closest to the DF curve. According to the matching degree between each particle group and the DF curve, the order from highest to lowest was S2 > S1 > S3 > S4 > S5.

#### 3.2.2. The Induced Nucleation Effect of SMR Powder in Cement Mortar

**Figure 13:**
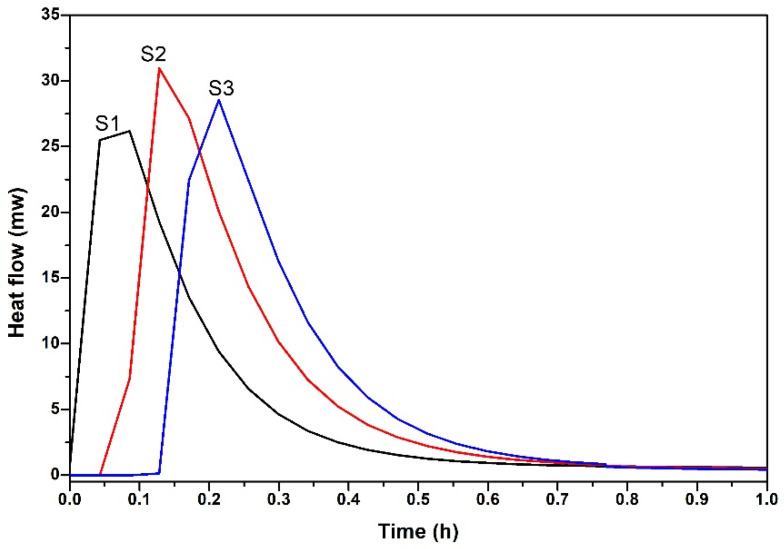
Heat flow of cementitious materials of S1, S2, and S3 after hydration for 1 h.

The position and peak height of the first exothermic peak during the hydration process of cement-based materials were often regarded as the important index to identify whether mineral admixtures had the induced nucleation effect [24]. In Section 3.1.2, the hydration characteristic of cementitious materials with SMR was studied. Figure 13 shows the heat flow curve of cementitious materials of S1, S2, and S3 after hydration for 1 h, which was extracted from Figure 8a. Compared with S1, the positions of the first heat flow peaks of S2 and S3 were successively delayed, but the height of the first heat flow peaks of S2 and S3 were successively increased. This indicated that the nucleation effect of SMR provided heterogeneous nucleation sites, which promote the initial hydrolysis of C_3_S and thereby increase the peak value of the first heat flow peak.

#### 3.2.3. The Pozzolanic Effect of SMR Powder in Cement Mortar

**Figure 14:**
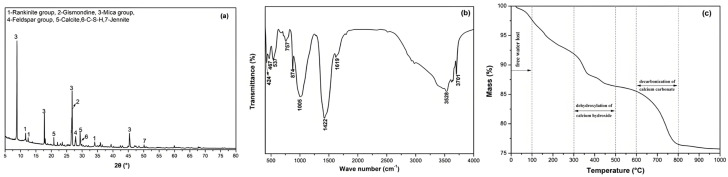
The XRD and FTIR analysis results of the products produced by simulation-pore solution alkali leaching SMR. (**a**) XRD pattern (**b**) FTIR pattern (**c**) TG curve [11].

SMR was soaked in simulation-pore solution, then, a white flocculent product produced on the surface of SMR particles. Figure 14a–c shows the XRD, FTIR, and TG analysis results of the white flocculent product, respectively.

In Figure 14a, the mineral phases of the white flocculent product were identified, including rankinite group, gismondine, mica group, feldspar group, calcite, C-S-H, and jennite. In Figure 14b, the FTIR spectra band at 467 cm^−1^ corresponded to the bending vibration of the Si-O [25]. Spectra bands in the region of 350 to 500 cm^−1^ and 700 to 950 cm^−1^ corresponded to the vibration of [AlO_4_] groups and [AlO_6_] groups, respectively. The [AlO_4_] and [AlO_6_] groups were derived from the gismondine and mica groups, respectively [26]. Bands at 713 cm^−1^, 875 cm^−1^, 1000 cm^−1^, and 1796 cm^−1^ corresponded to the vibration of CO_3_^2−^ in calcite. Bands in the region from 1600 cm^−1^ to 1800 cm^−1^ corresponded to the characteristic peak of the carbonyl group [27]. Bands in the region from 3200 cm^−1^ to 3600 cm^−1^ were matched to the bending and stretching vibrations of -OH, respectively. The symmetric stretching vibration of -OH in silicate was demonstrated by the spectra band at 3700 cm^−1^. In Figure 14c, with the increase of calcination temperature, four weightlessness zones were observed. The initial weight loss was caused by evaporation of free water. The weightlessness from 100 °C to 300 °C was inferred to be the bound water dehydration of C-S-H and C-(M)-S-H [28,29,30]. The weightlessness from 300 °C to 500 °C and 600 °C to 850 °C corresponded to the dehydroxylation of CH and the decomposition of calcite [31,32], respectively.

It was confirmed that SMR particles had weak pozzolanic activity in an alkaline environment. Early hydration of cement provided an alkaline environment for inducing Ca^2+^, Al^3+^, and Si^4+^ to leach out from the amorphous phase of SMR and produce C-S-H or its derivatives. During this process, the secondary hydration of SMR occurred. Part of the newly generated C-S-H adhered to the surface of SMR and filled the gap in the hardened specimen.

In summary, it was confirmed that the appropriate SMR incorporation into cement mortar could improve the structure and properties of cement-based materials through particle filling, the induced nucleation effect, and the pozzolanic effect.

### 3.3. The Life Cycle Assessment of Cement-Based Materials with SMR as Mineral Admixture

Green building materials are defined by the academic community as the processes that minimize resource consumption, have low energy consumption, and reduce ecological hazards through the entire life cycle from production to disposal [33]. In this research, the life cycle method was used for contrastive analysis of the environmental benefit of cement mortar with or without SMR to quantify the energy-saving potential of the comprehensive utilization of SMR in cement mortar.

The boundaries of the life cycle include the production of raw materials, the transportation of raw materials to the mixing station, the preparation of mortar, and the transportation of mortar to the construction site. The common data on the environmental impact involved in the calculation process is shown in Table 4. The mix ratio used for calculating environmental benefits is shown in Table 3. Table 5 shows the list of environmental impacts of the different raw material preparations.

Table 6 lists the environmental impact list of raw materials for preparation of 1 m^3^ cement mortar. It was not hard to find that the environmental impact of S1 was much more serious than the other mortar groups in which SMR as admixture partly replaced cement. The shipping distance of every raw material was set to 100 km and the environmental impact list of raw material transportation according to the environmental impact list of transportation equipment is calculated in Table 7. The energy consumption during the transportation stage of the raw materials used for producing 1 m^3^ mortar was 109 MJ. Further, the emissions of CO_2_, CH_4_, SO_2_, and NO_X_ were 29.2 kg, 0.136 kg, 0.0358 kg, and 0.88 kg, respectively.

Table 8 shows the environmental impact list of different stirring and curing methods. The blender with different capacities had different environmental impacts. The difference in environmental impacts between standard curing and steam curing was also significant. In this research, the blender with 3 m^3^ capacity was used. Additionally, the standard curing condition was used with the humidity at no less than 95% and the temperature set to 20 °C ± 2 °C. The environmental impacts of the stirring and the curing stage together constitute the environmental impact list for preblend stages shown in Table 8. The transportation distance is set to 200 km using a concrete mixer with 12 m^3^ capacity. The environmental impact list of cement mortar preparation and transportation is shown in Table 8. Table 9 listed the environmental impact list of the preparation and transportation stages under 1 m^3^ mortar.

**Figure 15:**
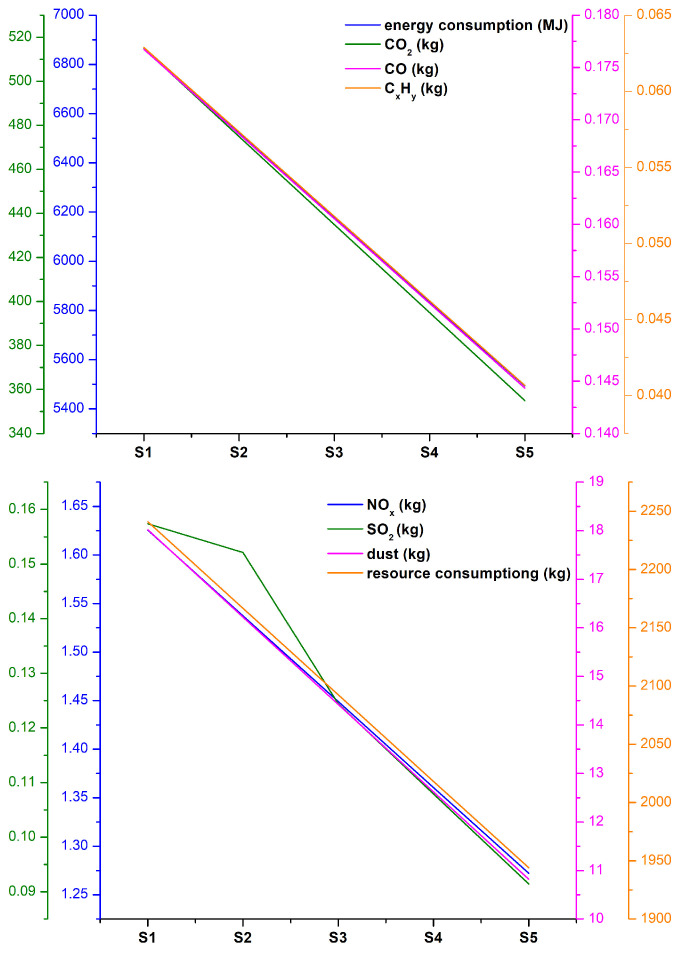
The environmental impact of cement mortars with or without SMR admixture.

In conjunction with the environmental impacts during the stages of raw material preparation, raw materials transportation, mortar mixing, mortar transportation, mortar molding, and mortar curing, the environmental impact list of the system boundary of the cement mortars was calculated and shown in Table 10 and Figure 15.

With the increase of SMR powder to replace cement, the environmental impact of cement mortar decreased. Using 1 m^3^ of cement mortar as an example, for every 10% increase in SMR powder replacing cement, the energy consumption decreased by 342 MJ, CO_2_ emissions were reduced by approximately 40 kg, CO emissions were reduced by 8.1 g, C_x_H_y_ emissions were reduced by 5.55 g, NO_x_ emissions were reduced by 88.3 g, SO_2_ emissions were reduced by 5.24 g, dust emissions were reduced by 1.80 kg, and resource consumption was reduced by 74.3 kg. When the content of SMR was less than 20 wt.%, the comprehensive performance of the cement mortar, including mechanical properties, durability, and workability, was similar to that of pure cement mortar. Based on unit comprehensive performance, the environmental emissions and resource consumption of the cement mortar with SMR admixture were much lower than those of pure cement mortar, which demonstrated excellent comprehensive benefits.

## 4. Conclusions

(1) The feasibility of SMR as mineral admixture in cement mortar was proved. The appropriate content of SMR could ensure the mechanical properties and durability of the cement mortar are similar to those of the blank group. SMR could improve workability and reduce the cumulative hydration heat release of cement mortar, which confirmed its application potential in large-scale pouring objects.

(2) The SMR particles with a low hydration activity index improved the structure and performance of cement mortar through particle filling, inducing nucleation and pozzolanic effects. This provided a reference for the application of low-activity mineral admixtures.

(3) The environmental impact of SMR as a mineral admixture in cement-based materials had been quantitatively calculated using the life cycle assessment method. The utilization of SMR could effectively reduce the environmental emissions and resource consumption of cement-based materials.

The results indicate that SMR is expected to be widely applied in cement-based materials and related engineering projects, and larger-scale experiments are necessary.

## Figures and Tables

**Table 1:** The chemical composition of raw materials (wt.%).

Raw Materials	SiO_2_	Al_2_O_3_	CaO	Fe_2_O_3_	K_2_O	Na_2_O	TiO_2_	MgO	SO_3_	P_2_O_3_	Others	LOI
SMR	66.17	16.06	5.64	5.21	3.10	1.13	0.87	0.79	0.27	0.22	0.23	0.32
CE	17.62	4.21	61.23	3.00	0.78	0.11	0.29	0.84	4.54	0.24	4.11	3.04
S	98.26	0.97	0.39	0.08	0.05	0.06	-	0.02	-	-	0.05	0.12

**Table 2:** The cumulative residue on sieve of standard sand.

Mesh size (mm)	2	1.6	1	0.5	0.16	0.08
Cumulative percentage remained (wt.%)	0	10	35	71	89	100

**Table 3:** The mix proportion of cement mortar with or without SMR.

Code	CE (g)	SMR Powder (g)	Cement Water Ratio (g)	Quartz Sand (g)
S1	450	0	0.5	1350
S2	405	45	0.5	1350
S3	360	90	0.5	1350
S4	315	135	0.5	1350
S5	270	180	0.5	1350

**Table 4:** Common data on the environmental impact of the common system.

Common System	Energy Consumption	CO_2_ (kg)	CO (g)	C_x_H_y_ (g)	NO_x_ (g)	SO_2_ (g)	Dust (kg)
Electricity (kW.h)	0.366 kg standard coal (10.7 MJ)	0.81	0.03	0.06	0.44	0.97	1.86 × 10^−3^
transportation (t.km^−1^)	diesel oil 0.2 L (7.14 MJ)	0.14	4.00	0.22	0.94	0.03	

**Table 5:** List of environmental impacts during the raw material production stage.

Raw Materials /t	Energy Consumption (MJ)	CO_2_ (kg)	CO (g)	C_x_H_y_ (g)	NO_x_ (g)	SO_2_ (g)	Dust (kg)	Resource Consumption (kg)
cement [34]	7610.00	886.00	26.80	35.70	1570.00	346.00	40.00	1650.00
water [35]	0.12	0.21	0.01	0.00	1.00	2.00	0.00	0.00
quartz sand [36]	776.00	60.40	0.00	2.33	4.67	0.00	0.02	1110.00
SMR powder [37]	9.88	0.86	0.03	1.68	3.09	1.21	0.00	0.00

**Table 6:** The environmental impact list of raw materials of cement mortar.

Cement Mortar	Energy Consumption (MJ)	CO_2_ (kg)	CO (g)	C_x_H_y_ (g)	NO_x_ (g)	SO_2_ (g)	Dust (kg)	Resource Consumption (kg)
S1	4470.00	480.00	12.10	19.20	713.00	156.00	18.00	2240.00
S2	4130.00	440.00	10.90	17.70	643.00	141.00	16.20	2170.00
S3	3790.00	401.00	9.65	16.10	572.00	125.00	14.40	2090.00
S4	3450.00	361.00	8.45	14.60	501.00	110.00	12.60	2020.00
S5	3100.00	321.00	7.24	13.10	431.00	94.10	10.80	1940.00

**Table 7:** Environmental impact list of transport machines.

Transport Machine (t. Km)	Energy Consumption (MJ)	CO_2_ (kg)	C_x_H_y_ (g)	SO_2_ (g)	NO_x_ (g)
Light-duty diesel truck, 2 t	1.32	0.21	1.63	0.43	2.74
Medium-sized diesel truck, 8 t	0.67	0.15	0.83	0.22	0.73
Heavy-duty diesel trucks, 10 t	0.61	0.16	0.76	0.20	4.89
Light-duty gasoline truck, 2 t	1.46	0.25	1.82	0.43	2.94
Medium-sized gasoline truck, 8 t	0.75	0.09	0.92	0.22	1.26
Heavy-duty gasoline truck, 10 t	6.73	0.12	0.82	0.20	2.01

**Table 8:** Environmental impact list of preparation stage under 1 m^3^ mortar [38].

Production Methods or Equipment		Energy Consumption (MJ)	CO_2_ (kg)	NO_x_ (g)	SO_2_ (g)	Dust (kg)
Stirring	blender 1.5 m^3^	16.30	0.70	0.24	0.29	0.05 × 10^−3^
	blender 2.0 m^3^	16.60	0.70	0.24	0.30	0.06 × 10^−3^
	blender 2.5 m^3^	13.50	0.60	0.20	0.24	0.05 × 10^−3^
	blender 3.0 m^3^	13.80	0.60	0.20	0.24	0.05 × 10^−3^
curing	standard curing	0.00	0.00	0.00	0.00	0.00 × 10^−3^
	steam curing	593.00	38.50	24.10	31.70	34.80 × 10^−3^
	high pressure steam curing	712.00	46.20	28.90	38.10	41.70 × 10^−3^

**Table 9:** Environmental impact list of the preparation and transportation stages under 1 m^3^ mortar.

Life Stage /1 m^3^	Energy Consumption (MJ)	CO_2_ (kg)	CO (g)	C_x_H_y_ (g)	NO_x_ (g)	SO_2_ (g)	Dust (kg)
Preblend	13.80	0.60	0.00	0.00	0.20	0.24	0.05 × 10^−3^
Transportation	2280.00	4.81	28.60	7.85	32.10	0.97	0.00 × 10^−3^
total	2280.00	5.41	28.60	7.85	32.30	1.22	0.05 × 10^−3^

**Table 10:** The list of environmental impacts of system boundaries of cement mortars.

Cement Mortar /1 m^3^	Life Stage	Energy Consumption (MJ)	CO_2_ (kg)	CO (g)	C_x_H_y_ (g)	NO_x_ (g)	SO_2_ (g)	Dust (kg)	Resource Consumption (kg)
S1	raw materials preparation	4470.0	480.0	12.1	19.2	713.0	156.0	18.0	2240.0
	raw materials transportation	109.0	29.2	136.0	35.8	880.0	0.0	0.0	0.0
	cement mortar preparation	2280.0	5.4	28.6	7.9	32.3	1.2	0.0	0.0
	boundary systems	6860.0	515.0	177.0	62.9	1630.0	157.0	18.0	2240.0
S2	raw materials preparation	4130.0	440.0	10.9	17.7	643.0	141.0	16.2	2170.0
	raw materials transportation	109.0	29.2	136.0	35.8	880.0	32.8	0.0	0.0
	cement mortar preparation	2280.0	5.4	28.6	7.9	32.3	1.2	0.0	0.0
	Solid waste utilization	0.0	−0.2	−6.9	−4.0	−17.8	−1.0	0.0	0.0
	boundary systems	6520.0	475.0	169.0	57.3	1540.0	152.0	16.2	2170.0
S3	raw materials preparation	3790.0	401.0	9.7	16.1	572.0	125.0	14.4	2090.0
	raw materials transportation	109.0	29.2	136.0	35.8	880.0	0.0	0.0	0.0
	cement mortar preparation	2280.0	5.4	28.6	7.9	32.3	1.2	0.0	0.0
	Solid waste utilization	−0.1	−0.3	−13.8	−8.1	−35.6	−1.9	0.0	0.0
	boundary systems	6180.0	435.0	161.0	51.8	1450.0	124.0	14.4	2090.0
S4	raw materials preparation	3450.0	361.0	8.5	14.6	501.0	110.0	12.6	2020.0
	raw materials transportation	109.0	29.2	136.0	35.8	880.0	0.0	0.0	0.0
	cement mortar preparation	2280.0	5.4	28.6	7.9	32.3	1.2	0.0	0.0
	Solid waste utilization	−0.1	−0.5	−20.7	−12.1	−53.4	−2.9	0.0	0.0
	boundary systems	5840.0	395.0	152.0	46.2	1360.0	108.0	12.6	2020.0
S5	raw materials preparation	3100.0	321.0	7.2	13.1	431.0	94.1	10.8	1940.0
	raw materials transportation	109.0	29.2	136.0	35.8	880.0	0.0	0.0	0.0
	cement mortar preparation	2280.0	5.4	28.6	7.9	32.3	1.2	0.0	0.0
	Solid waste utilization	−0.2	−0.6	−27.6	−16.1	−71.2	−3.9	0.0	0.0
	boundary systems	5490.0	355.0	144.0	40.7	1270.0	91.4	10.8	1940.0

## Data Availability

The original contributions presented in this study are included in the article. Further inquiries can be directed to the corresponding authors.
